# High prevalence of *Clonorchis sinensis* infection in Guangxi, Southern China

**DOI:** 10.1186/s41182-021-00297-0

**Published:** 2021-01-19

**Authors:** Zhi-Hua Jiang, Xiao-Ling Wan, Guo-Li Lv, Wei-Wei Zhang, Yuan Lin, Wen-Qian Tang, Hai-Yan Wei, Fang-Qi Ou, Yun-Liang Shi, Yi-Chao Yang, Jian Liu, Carlos H. F. Chan

**Affiliations:** 1 Guangxi Zhuang Autonomous Region Center for Disease Prevention and Control, 18 Jin Zhou Road, Nanning, 530028 Guangxi China; 2 University of Iowa Carver College of Medicine, 200 Hawkins Drive, JCP 4642, Iowa City, IA 52242 USA

**Keywords:** Soil-transmitted helminths (STHs), *Clonorchis sinensis*, Prevalence, Epidemiology

## Abstract

**Background:**

Soil-transmitted helminths (STHs), such as hookworm, roundworm and whipworm, and food-borne trematodiases, including *Clonorchis sinensis*, remain a public health problem worldwide, especially in tropical and subtropical regions.

**Objective:**

We aimed to determine the current prevalence of these parasites in Guangxi, China, which is located in a subtropical region.

**Methods:**

A cross-sectional study and a 4-year longitudinal surveillance study were carried out. Stool samples were collected and examined microscopically for parasite eggs using the modified Kato-Katz thick smear method.

**Results:**

The study subjects selected using stratified random cluster sampling for the cross-sectional study and longitudinal surveillance study numbered 15,683 and 24,429, respectively. In the cross-sectional study, hookworm, roundworm, whipworm, pinworm, *C. sinensis*, and tapeworm were found. The total prevalence of soil-transmitted helminths (STHs) was 6.4% (95% CI, 6.0-6.8). The prevalences of *C. sinensis*, hookworm, roundworm, whipworm, and pinworm were 10.6%, 4.2%, 0.3%, 0.3%, and 1.8%, respectively. The prevalence of *C. sinensis* in males (14.0%, 95% CI, 13.3-14.8) was significantly higher than in females (7.2%, 95% CI, 6.7-7.8) (*P* = 0.0001). The prevalence also was significantly higher in the medical worker group (20.8%, 95% CI, 12.9-28.7) than in all other occupational groups (10.5%, 95% CI, 10.0-11.0) (*P* = 0.0001). The prevalence of hookworm in females (5.3%, 95% CI, 4.8-5.8) was significantly higher than in males (3.0%, 95% CI, 2.6-3.3) (*P* = 0.0001). In the longitudinal surveillance study, the prevalence of *C. sinensis* and STHs in 2016, 2017, 2018, and 2019 were 12.0%, 6.0%, 11.0%, and 10.0% and 2.6%, 2.8%, 1.5%, and 1.5%, respectively.

**Conclusions:**

Adult male and occupation of and medical workers are risk factors for infection with *C. sinensis* and hookworm. The prevalence rate of *C. sinensis* remains high while those of the other STHs are decreasing, suggesting that enhanced health education should be focused on *C. sinensis* in Guangxi.

## Background

There was a downward trend in the global prevalence and incidence of human parasitic diseases between 1990 and 2016 [1]. Meanwhile, the prevalence rates of several parasitic infections have actually increased substantially since 1990, including food-borne trematodiases and soil-transmitted helminth infections. Some of these parasitic diseases are among the most prevalent neglected tropical diseases worldwide [1, 2].

Soil-transmitted helminths (STHs), which can cause helminthiasis, include hookworm (*Necator americanus* and *Ancylostoma duodenale*), roundworm (*Ascaris lumbricoides*), and whipworm (*Trichuris trichiura*) [3]. Globally, 1.5 billion people are infected with STHs [4]. These infections are widely distributed in tropical and subtropical areas, with the greatest numbers occurring in sub-Saharan Africa, the Americas, China, and East Asia [4].

Food-borne trematodiases, including liver flukes, lung flukes and intestinal flukes, pose significant public health and economic problems, yet these diseases are often neglected [5]. *Clonorchis sinensis* (*C. sinensis*), the oriental liver fluke, is endemic in parts of Asia, including China, Japan, Korea, and Vietnam. Currently, it is estimated that more than 200 million people are at risk of *C. sinensis* infection, and over 15 million are infected worldwide [6]. The parasite causes a substantial clinical or subclinical disease, known as clonorchiasis [6, 7].

The prevalence of STHs in a specific country depends on environmental, socioeconomic, and demographic factors, including the health-related behavior of the population and access to hygienic latrines and to treated water [8]. Eating raw food can lead to infection with food-borne parasites and barefooted agricultural work can lead to hookworm infection [9].

China is positioned across tropical, subtropical, temperate, and cold zones, so that the prevalence of STHs varies among the provinces [2]. Guangxi Zhuang autonomous region has a subtropical climate that is suitable for the survival and reproduction of human parasites [10]. Furthermore, inhabitants in Guangxi have a tradition of eating raw fish. More and more people can afford to eat raw fish with the development of the economy, which increases the risk of infection with liver flukes. Therefore, regular surveillance is important for determining policies to eliminate these parasites in Guangxi. The objective of this study was to carry out a cross-sectional study and a longitudinal surveillance study with the purpose of clarifying the current prevalence of these important human parasites in Guangxi.

## Materials and methods

### Study population and sample collection

All study subjects were recruited from a rural area of Guangxi, China. The cross-sectional studies were carried out in 2019. Three counties were selected randomly from the east, south, west, north, and center of Guangxi. Then, one town was selected randomly from the east, south, west, north, and center of each selected county. Finally, one administrative village was selected randomly from each town and 200 inhabitants aged over three were selected from each village using random cluster sampling. In total, 1000 inhabitants were recruited from each county.

The longitudinal surveillance study was established in 2016 and follow-up once every year until 2019. Two counties were first selected from a hyperendemic area (infection rate > 10%), endemic area (infection rate between 1 and 10%), and low prevalence area (infection rate < 1%). Then, one town was selected from the east, south, west, north, and center of each selected county. Finally, one administrative village was selected randomly from each town and 200 inhabitants aged over three were selected from each village using random cluster sampling. In total, 1000 inhabitants were recruited from each county. We carried out surveillance in the selected village annually.

To raise awareness of this survey, advertisements and several promotional events were organized, such as posters in public locations and sending each family a letter stating the objectives of the survey, prior to starting recruitment. Each study subject completed a one-page questionnaire and provided a fecal sample of 50 grams for the measurement of parasite eggs. Stool examination was provided free of charge to all interested individuals.

The study team comprises doctors from the Centres for Disease Prevention and Control of Guangxi Province and local counties and all investigators were trained before the investigation. From 2016, the study teams traveled to each village in the selected counties, accompanied by village doctors, to visit and inform all inhabitants, and obtain a 50-g sample of feces for stool examination. A one-page questionnaire was completed by the investigator to record basic information, which included gender, date of birth, ethnicity, and history of eating raw fish, by face-to-face interview with the study subject or the parent (for children < 18 years of age).

### Stool examination

Stool samples (> 30 g) were examined microscopically for parasite eggs using the modified Kato-Katz thick smear method (two slides for each sample) [11]. Samples positive for hookworm were cultured to differentiate *A. duodenale* and *N. americanus.* For each county, 50 stool samples positive for hookworm were cultured (all were cultured if fewer than fifty samples were positive) using the culture method [2]. In brief, 0.5 g from each sample was smeared on a filter paper, which was put into a tube with water so that the water level reached the filter paper but not the stool sample. The tube was then kept in a moist atmosphere at 31 °C for 4 days or 26 ~ 30^o^C for 6 ~ 8 days. After this period of culture, each sample was dipped into water in a beaker at 45 °C for 1 h to allow potential hookworm larvae to emerge. The supernatant was carefully poured out after 1 h, keeping 0.5 ml water with the larvae at the bottom of the beaker. Children aged three to nine were also examined for the eggs of *Enterobius vermicularis* using the cellophane-tape perianal swab method [12]. All parasite eggs of each species were identified according to morphology [13]. To control accuracy, 10% of the positive stool samples and 5% of the negative samples were re-examined by a senior technician for quality assurance. Discrepancies were resolved by a third reader to determine the final egg count. The number of eggs identified per slide was multiplied by 24 and recorded as the egg count per gram of feces (EPG).

### Definitions

The intensities of helminth infections were expressed in terms of egg count per gram feces (EPG). Ascaris lumbricoides: light 1-4999 EPG; moderate 5000-49,999 EPG; heavy > 50,000 EPG. Trichuris trichiura: light 1-999 EPG; moderate 1000-9999 EPG; heavy > 10,000 EPG. Hookworms: light 1-1999 EPG; moderate 2000-3999 EPG; heavy > 4000 EPG [14]. The infection intensity of *C. sinensis* was categorized as light (1–999 EPG), moderate (1000–9999 EPG), and heavy (≥ 10,000 EPG) [15].

### Statistical analysis

Statistical comparisons of the prevalence of overall and separate species of parasites between males and females and across the different age groups and different areas were performed using chi-squared tests. All *P* values were two-tailed and *P* < 0.05 was considered to be significant. The 95% confidence interval (CI) for the prevalence of parasites was estimated. Statistical analysis was carried out using EPI info version 6.1 and the SPSS version 19.0 software.

## Results

### Data of STHs and *C. sinensis* infection from the cross-sectional study

#### General information

In total, 15 counties were surveyed and 15,683 inhabitants were recruited, 7704 males and 7979 females. Their average ages were 38.9 ± 22.9 (SD) and 40.8 ± 22.9 (SD) years, respectively. Hookworm, roundworm, whipworm, pinworm, *C. sinensis*, and tapeworm were found. However, tapeworm was found in one inhabitant only and was excluded from the analysis.

The total prevalence of STHs was 6.4% (95% CI, 6.0-6.8). The prevalence in males (5.3%, 95% CI, 4.8-5.8) was significantly lower than that in females (7.5%, 95% CI, 6.9-8.0) (*P* = 0.0001). The prevalences of *C. sinensis*, hookworm, roundworm, whipworm, and pinworm were 10.6%, 4.2%, 0.3%, 0.3%, and 1.8%, respectively (Table 1). The proportions of Necator americanus and Ancylostoma duodenale were 97.5% (154/158) and 2.5% (4/158), respectively.

**Table 1:** The prevalence of STHs^a^ and *C. sinensis* according to geographic region in Guangxi^b^

Regions	Number	*C. sinensis*		Hookworm		Roundworm		Whipworm		Pinworm		Total of STHs	
		N. positive	Prevalence (%) (95% CI^c^)	N. positive	Prevalence (%) (95% CI)	N. positive	Prevalence (%) (95% CI)	N. positive	Prevalence (%) (95% CI)	N. positive	Prevalence (%) (95% CI)	N. positive^d^	Prevalence (%) (95% CI)
East	3248	384	11.8 (10.7-12.9)	87	2.7 (2.1-3.2)	16	0.5 (0.3-0.7)	11	0.3 (0.1-0.5)	26	0.8 (0.5-1.1)	138	4.3 (3.6-4.9)
South	3133	55	1.8 (1.3-2.2)	346	11.0 (9.9-12.1)	0	0.0	5	0.2 (0.02-0.3)	62	2.0 (1.5-2.57)	409	13.1 (11.9-14.2)
West	3138	14	0.5 (0.2-0.7)	65	2.1 (1.6-2.6)	25	0.8 (0.5-1.1)	17	0.5 (0.3-0.8)	8	0.3 (0.08-0.4)	113	3.6 (3.0-4.3)
North	3113	563	18.1 (16.7-19.4)	114	37 (3.0-4.3)	0	0.0	11	0.4 (0.1-0.6)	48	1.5 (1.1-2.0)	172	5.5 (4.7-6.3)
Center	3051	643	21.1 (19.6-22.5)	40	1.3 (0. 1-1.7)	0	0.0	1	0.03 (−0.03-0.1)	133	4.4 (3.6-5.1)	174	5.7 (4.9-6.5)
Total	15683	1659	10.6 (10.1-11.1)	652	4.2 (3.9-4.5)	41	0.3 (0.2-0.3)	45	0.3 (0.2-0.4)	277	1.8 (1.6-2.0)	1006	6.4 (6.0-6.8)

^a^*STHs* soil-transmitted helminths

^b^Data from the cross-sectional study

^c^*CI* confidence interval

^d^Some of these are dual or triple parasitic infections, so the total number of STHs is underrepresented

#### The prevalence of STHs and *C. sinensis* according to geographic region

The prevalence of STHs in the southern region of Guangxi is highest (13.1%, 95% CI, 11.9-14.2), and that in the west is the lowest (3.6%, 95% CI, 2.9-4.3). The difference is significant (*P* = 0.0001). The prevalence of *C. sinensis* in the center is highest (21.1%, 95% CI, 19.6-22.5), and that in the west is the lowest (0.5%, 95% CI, 0.2-0.7). Again, the difference is significant (*P* = 0.0001). The highest prevalences of hookworm, roundworm, whipworm, and pinworm were seen in the south (11.0%, 95% CI, 9.9-12.1), West (0.8%, 95% CI, 0.5-1.1), west (0.5%, 95% CI, 0.3-0.8), and center (4.4%, 95% CI, 3.6-5.1), respectively. Clearly, the prevalence of STHs and *C. sinensis* varies geographically (Table 1).

#### The risk factors of STHs and *C. sinensis* infection

The prevalence of *C. sinensis* in males (14.0%, 95% CI, 13.3-14.8) was significantly higher than in females (7.2%, 95% CI, 6.7-7.8) (*P* = 0.0001). The prevalence of hookworm in females (5.3%, 95% CI, 4.8-5.8) was significantly higher than in males (3.0%, 95% CI, 2.6-3.3) (*P* = 0.0001). The prevalence of roundworm, whipworm, and pinworm did not differ significantly between males and females (Table 2).

**Table 2:** Distribution of *C. sinensis* and STHs^a^ according to socio-demographic characteristics in cross-sectional study

Risk factors		Number	*C. sinensis*		Hookworm		Roundworm		Whipworm		Pinworm	
			N. positive	Prevalence(%) (95% CI^b^)	N. positive	Prevalence(%) (95% CI)	N. positive	Prevalence(%) (95% CI)	N. positive	Prevalence(%) (95% CI)	N. positive	Prevalence(%) (95% CI)
Gender	Male	7704	1082	14.0 (13.3-14.8)	228	3.0 (2.6-3.3)	21	0.3 (0.2-0.4)	22	0.3 (0.2-0.4)	144	1.9 (1.6-2.2)
	Female	7979	577	7.2 (6.7-7.8)	424	5.3 (4.8-5.8)	20	0.3 (0.1-0.4)	23	0.3 (0.2-0.4)	133	1.7 (1.4-2.0)
Education	Pre-school	1596	14	0.9 (0.4-1.3)	10	0.6 (0.2-1.0)	9	0.6 (0.2-0.9)	4	0.3 (0.0-0.5)	155	9.7 (8.3-11.2)
	Illiterate	1158	94	8.1 (6.6-9.7)	93	8.0 (6.5-9.6)	0	0.0	2	0.2 (− 0.1-0.4)	0	0.0
	Primary school	5980	439	7.3 (6.7-8.0)	320	5.4 (4.8-5.9)	20	0.3 (0.2-0.5)	20	0.3 (0.2-0.5)	113	1.9 (1.5-2.2)
	Middle school	6663	1080	16.2 (15.3-17.1)	227	3.4 (3.0-3.9)	12	0.2 (0.1-0.3)	19	0.3 (0.2-0.4)	9	0.1 (0.1-0.2)
	University	286	32	11.2 (7.5-14.8)	2	0.7 (−0.3-1.7)	0	0.0	0	0.0	0	0.0
Occupation	Pre-school	1568	14	0.9 (0.4-1.4)	9	0.6 (0.2-0.9)	9	0.6 (0.2-0.9)	4	0.3 (0.0-0.5)	155	9.9 (8.4-11.4)
	Students	2359	52	2.2 (1.6-2.8)	19	0.8 (0.5-1.2)	9	0.4 (0.1-0.6)	3	0.1 (−0.0-0.3)	109	4.6 (3.8-5.5)
	Teachers	115	12	10.4 (4.8-16.0)	2	1.7 (−0.7-4.1)	0	0.0	0	0.0	0	0.0
	Medical workers	101	21	20.8 (12.9-28.7)	2	2.0 (− 0.7-4.7)	0	0.0	0	0.0	0	0.0
	Company workers	115	10	8.7 (3.6-13.9)	1	0.9 (−0.83-2.57)	0	0.0	1	0.9 (-0.8-2.6)	0	0.0
	Agricultural workers	11319	1529	13.5 (12.9-14.1)	618	5.5 (5.0-5.9)	23	0.20 (0.1-0.3)	36	0.3 (0.2-0.4)	13	0.1 (0.1-0.2)
	Others^b^	106	21	19.8 (12.2-27.4)	1	0.9 (-0.9-2.8)	0	0.0	1	0.9 (−0.9-2.8)	0	0.0
Ethnicity	Han	7596	488	6.4 (5.9-7.0)	202	2.7 (2.3-3.0)	24	0.3 (0.2-0.5)	25	0.3 (0.2-0.5)	85	1.1 (0.9-1.4)
	Zhuang	6987	736	10.5 (9.8-11.3)	395	5.7 (5.1-6.2)	3	0.0 (-0.0-0.1)	9	0.1 (0.1-0.2)	183	2.6 (2.3-3.0)
	Miao	105	73	69.5 (60.7-78.3)	11	10.5 (4.6-16.3)	0	0.0	0	0.0	0	0.0
	Dong	579	358	61.8 (57.9-65.8)	12	2.1 (0.9-3.2)	0	0.0	3	0.5 (−0.1-1.1)	7	1.2 (0.3-2.1)
	Yao	391	4	1.0 (0.0-2.0)	31	7.9 (5.3-10.6)	14	3.6 (1.7-5.4)	8	2.1 (0.7-3.5)	2	0.5 (− 0.2-1.2)
	Others	25	0	0.0	1	4.0 (−3.7-11.7)	0	0.0	0	0.0	0	0.0

^a^*STHs* soil-transmitted helminths

^b^*CI* confidence interval

In general, the prevalence of *C. sinensis* increases dramatically from the age of 10 years. In contrast, the prevalence of pinworm decreases dramatically after that age. The prevalence of hookworm increases with age; its prevalence among those aged over 50 years (7.0%, 95% CI, 6.4-7.6) is significantly higher than that of those below 50 years old (2.2%, 95% CI, 1.9-2.5) (*P* = 0.0001). No significant change with age was found in the prevalence of roundworm and whipworm (Table 3).

**Table 3:** Age-dependent prevalence of the various parasites in Guangxi

Ages	*C. sinensis*^a^			*N*	*C. sinensis*^b^		Hookworm^b^		Roundworm^b^		Whipworm^b^		Pinworm^b^	
	*N*	N. positive	Prevalence(%) (95% CI^c^)		N. positive	Prevalence(%) (95% CI)	N. positive	Prevalence(%) (95% CI)	N. positive	Prevalence(%) (95% CI)	N. positive	Prevalence(%) (95% CI)	N. positive	Prevalence(%) (95% CI)
0-	4815	67	1.4 (1.1-1.7)	2764	28	1.0 (0.6-1.4)	19	0.69 (0.4-1.0)	15	0.5 (0.3-0.8)	5	0.2 (0.0-0.3)	262	9.5 (8.39-10.57)
10-	3311	63	1.9 (1.4-2.4)	1209	40	3.3 (2.3-4.3)	9	0.74 (0.3-1.2)	3	0.3 (−0.0-0.5)	2	0.2 (− 0.16-0.4)	2	0.2 (− 0.1-0.4)
20-	1615	159	9.9 (8.4-11.3)	971	131	13.5 (11.3-15.6)	18	1.85 (1.0-2.7)	0	0.0	1	0.1 (−0.1-0.3)	1	0.1 (−0.1-0.3)
30-	2903	385	13.3 (12.0 − 14.5)	1947	293	15.1 (13.5 − 16.6)	57	2.93 (2.2 − 3.7)	7	0.4 (0.1 − 0.6)	9	0.5 (0.2 − 0.8)	2	0.1 (−0.0-0.2)
40-	3402	532	15.6 (14.4-16.9)	2412	380	15.8 (14.3-17.2)	102	4.23 (3.4-5.0)	8	0.3 (0.1-0.6)	10	0.4 (0.2-0.7)	3	0.1 (−0.0-0.3)
50-	3960	664	16.8 (15.6-17.9)	2807	411	14.6 (13.3-16.0)	171	6.1 (5.21-7.0)	2	01 (−0.0-0.2)	7	0.3 (0.1-0.4)	1	0.0 (−0.0-0.1)
60-	3151	395	12.5 (11.4-13.7)	2154	237	11.0 (9.7-12.3)	165	7.7 (6.54-8.8)	2	0.1 (−0.0-0.2)	8	0.4 (0.1-0.6)	5	0.2 (0.0-0.4)
70-	1784	199	11.2 (9.7-12.6)	1097	109	9.9 (8.2-11.7)	83	7.57 (6.0-9.1)	2	0.2 (−0.1-0.4)	2	0.2 (−0.1-0.4)	0	0.0
80-	488	36	7.4 (5.1-9.7)	322	30	9.3 (6.1-12.5)	28	8.7 (5.6-11.8)	2	0.6 (−0.2-1.5)	1	0.3 (−0.3-0.9)	1	0.3 (−0.3-0.9)

^a^Data from the longitudinal surveillance study

^b^Data from the cross-sectional study

^c^*CI* confidence interval

According to education levels, the highest prevalences of *C. sinensis*, hookworm, roundworm, whipworm, and pinworm were seen in middle school, illiterates, pre-school, primary school, and pre-school, respectively. The prevalence of *C. sinensis* in middle school (16.2%, 95% CI, 15.3-17.1) was significantly higher than that of university students (11.2%, 95% CI, 7.5-14.8) (*P* = 0.023). The prevalence of hookworm in illiterates (8.0%, 95% CI, 6.5-9.6) is significantly higher than that of primary school (5.4%, 95% CI, 4.8-5.9) (*P* = 0.0001). The prevalence of pinworm of pre-school is also significantly higher than that of primary school (*P* = 0.0001).

The prevalence of *C. sinensis* in the medical worker group (20.8%, 95% CI, 12.9-28.7) was significantly higher than in all other occupations (10.5%, 95% CI, 10.0-11.0) (*P* = 0.0001). The prevalence of hookworm in agricultural workers is higher than all other occupations (*P* = 0.0001) (Table 2).

The prevalence of hookworm among individuals of Han ethnicity (2.7%, 95% CI, 2.3-3.0) was significantly lower than that of the ethnic minorities (5.6%, 95% CI, 5.1-6.1) (*P* = 0.0001). The prevalence of pinworm among individuals of Han ethnicity (1.1%, 95% CI, 0.9-1.4) was significantly lower than that of the ethnic minorities (2.4%, 95% CI, 2.0-2.7) (*P* = 0.0001). The prevalence of roundworm and whipworm did not differ significantly between Han and the ethnic minorities (Table 2).

Clearly, gender and occupation are associated with the prevalence of *C. sinensis* and hookworm. Age is associated with the prevalence of *C. sinensis*, hookworm, and pinworm. While belonging to an ethnic minority is associated with the infection of hookworm and pinworm.

#### The prevalence and intensity of STHs and *C. sinensis* according to the species of parasite

The average infection intensity with the various species of parasites was relatively light. The proportion of heavy infection with *C. sinensis* (4.3%, 72/1659, 95% CI, 0.4-0.6) was significantly higher than that of STHs (2.0%, 15/738, 95% CI, 1.0-3.0) (*P* = 0.005) (Table 4).

**Table 4:** The prevalence and intensity of STHs and *C. sinensis* according to the parasite species in the cross-sectional study

Species of parasite	Total			Light load			Moderate load			Heavy load		
	Number	N. positive	Prevalence(%) (95% CI^b^)	Number	Prevalence(%) (95% CI)	Proportion^a^ (%)	Number	Prevalence(%) (95% CI)	Proportion (%)	Number	Prevalence(%) (95% CI)	Proportion (%)
*C. sinensis*^a^	15683	1659	10.6 (10.1-11.1)	1209	7.7 (7.3-8.1)	72.9	378	2.4 (2.2-2.7)	22.8	72	0.5 (0.4-0.6)	4.3
Hookworm	15683	652	4.2 (3.9-4.5)	627	4.0 (3.7-4.3)	96.2	12	0.1 (0.0-0.1)	1.8	13	0.1 (0.0-0.1)	2.0
Roundworm	15683	41	0.3 (0.2-0.3)	28	0.2 (0.1-0.3)	68.3	11	0.0 (0.0-0.1)	26.8	2	0.0 (− 0.0-0.0)	4.9
Whipworm	15683	45	0.3 (0.2-0.4)	44	0.3 (0.2-0.4)	97.8	1	0.0 (−0.0-0.0)	2.2	0	0.0	0.090

^a^Proportion is the fraction of the total number of those positive for the same parasite

^b^*CI* confidence interval

#### Mix-infection of STHs and *C. sinensis* infection

In the cross-sectional study, the rate of dual parasitic infection was 0.2-0.4% and the triple infection rate was 0.004-0.01%. Dual infection with *C. sinensis* and hookworm (0.3%, 95% CI, 0.2-0.3) is significantly more common than any other double infection (0.1%, 95% CI, 0.1-0.2), according to the cross-sectional study (*P* = 0.02) (Table 5).

**Table 5:** Patterns of STHs and *C. sinensis* infections among 15,683 subjects from the cross-sectional study

Species	Positive number	Prevalence (%)
Dual parasitic infection		
*C. sinensis* + hookworm	39	0.3 (0.2-0.3)
*C. sinensis* + roundworm	4	0.03 (0.0-0.1)
*C. sinensis* + whipworm	5	0.03 (0.0-0.1)
*C. sinensis* + pinworm	4	0.03 (0.0-0.1)
Hookworm + roundworm	1	0.01 (− 0.01-0.03)
Hookworm + whipworm	1	0.01(− 0.01-0.03)
Hookworm + pinworm	2	0.01 (− 0.01-0.03)
Roundworm + whipworm	3	0.02 (0.00-0.04)
Whipworm + pinworm	1	0.01 (− 0.01-0.03)
Total	60	0.4 (0.3-0.5)
Triple parasitic infection		
*C. sinensis* + roundworm + whipworm	1	0.01 (− 0.01-0.03)

### Data of STHs and *C. sinensis* infection from the longitudinal surveillance study

#### General information

Six counties were included and 24,429 inhabitants were surveyed, 12,459 males and 11970 females. Their averages age were 36.5 ± 23.5 and 39.1 ± 23.5 years, respectively. *Clonorchis sinensis*, hookworm, roundworm, whipworm, and pinworm were found.

#### The prevalence of STHs

The total prevalence of hookworm, roundworm, whipworm, and pinworm were 1.5% (95% CI, 1.3-1.7), 0.1% (95% CI, 0.1-0.1), 0.3% (95% CI, 0.2-0.3), 0.2 (95% CI, 0.2-0.3), respectively (Table 6). The prevalence of STHs, including hookworm, roundworm, whipworm, and pinworm, in 2016, 2017, 2018, and 2019 were 2.6% (95% CI, 2.2-3.0), 2.8% (95% CI, 2.4-3.3), 1.5% (95% CI, 1.2-1.8), and 1.5% (95% CI, 1.2-1.8), respectively. The prevalence rate tends to be decreasing.

**Table 6:** The prevalence of *C. sinensis* and STHs from the longitudinal surveillance study

		Number	*C. sinensis*		Hookworm		Roundworm		Whipworm		Pinworm	
			N. positive	Prevalence (%) (95% CI^a^)	N. positive	Prevalence (%) (95% CI)	N. positive	Prevalence (%) (95% CI)	N. positive	Prevalence (%) (95% CI)	N. positive	Prevalence (%) (95% CI)
Hyperendemic area												
	Binyang	4606	875	19.0 (17.9-20.1)	7	0.2 (0.04-0.3)	0	0.0	4	0.1 (0.00-0.2)	6	0.1 (0.0-0.2)
	Hengxian	3033	988	32.6 (30.9-34.2)	6	0.2 (0.0-0.4)	1	0.0 (−0.0-0.1)	3	0.1 (− 0.0-0.2)	5	0.2 (0.0-0.3)
	Total	7639	1863	24.4 (23.4-25.4)	13	0.2 (0.1-0.3)	1	0.0 (−0.0-0.0)	7	0.1 (0.0-0.2)	11	0.1 (0.1-0.2)
Endemic area												
	Lingshan	4449	212	4.8 (4.2-5.4)	29	0.7 (0.4-0.9)	13	0.3 (0.1-0.5)	5	0.1 (0.0-0.2)	5	0.1 (0.0-0.2)
	Tianyang	4155	312	7.5 (6.7-8.3)	212	5.1 (4.3-5.8)	0	0.0	34	0.8 (0.5-1.1)	3	0.1 (−0.0-0.2)
	Total	8604	524	6.1 (5.6-6.6)	241	2.8 (2.5-3.1)	13	0.2 (0.1-0.2)	39	0.5 (0.3-0.6)	8	0.1 (0.0-0.2)
Low prevalence area												
	Jingxi	4023	17	0.4 (0.2-0.6)	79	2.0 (0.0-0.3)	10	0.3 (0.1-0.4)	8	0.2 (0.1-0.3)	11	0.3 (0.1-0.4)
	Qinnan	4163	22	0.5 (0.3-0.7)	34	0.8 (0.5-1.1)	0	0.0	13	0.3 (0.1-0.5)	20	0.5 (0.3-0.7)
	Total	8186	39	0.5 (0.3-0.6)	113	1.4 (1.1-1.6)	10	0.1 (0.6-0.2)	21	0.3 (0.2-0.4)	31	0.4 (0.2-0.5)
Total		24429	2426	9.9 (9.6-10.3)	367	1.5 (1.3-1.7)	24	0.1 (0.1-0.1)	67	0.3 (0.2-0.3)	50	0.2 (0.1-0.3)

^a^*CI* confidence interval

#### The prevalence of *C. sinensis*

The total prevalence of *C. sinensis* was 9.9% (2426/24429, 95% CI, 9.6-10.3). The prevalence in males (14.6%, 95 CI, 14.0-15.2) was significantly higher than in females (5.1%, 95% CI, 4.7-5.4) (*P* = 0.005). The total prevalence of *C. sinensis* in the hyperendemic, endemic, and low prevalence areas were 24.4%, 6.1%, and 0.4%, respectively (Table 6).

**Fig. 1:**
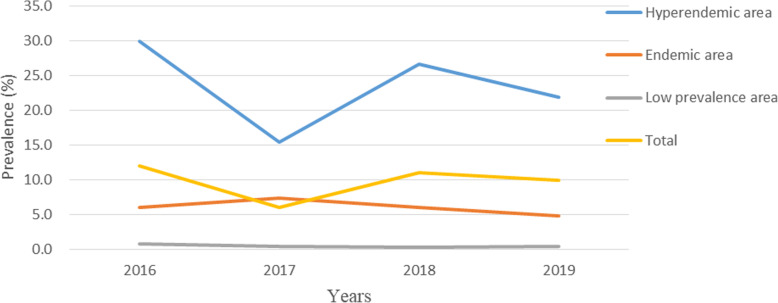
Trend of the prevalence of *C. sinensis* in Guangxi

**Fig. 2:**
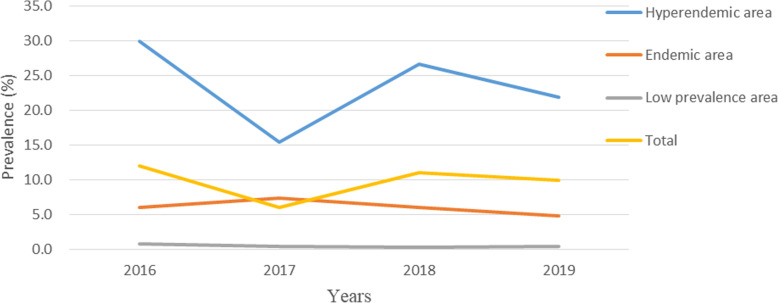
The trend of the prevalence of STHs in various counties

The total prevalence of *C. sinensis* in 2016, 2017, 2018, and 2019 were 12.0% (95% CI, 11.6-12.4), 6.0% (95% CI, 5.7-6.3), 11.0% (95% CI, 10.6-11.4), and 10.0% (95% CI, 9.6-10.4), respectively. Clearly, the prevalence rate of *C. sinensis* remains high (Fig. 1). The prevalence of *C. sinensis* in Hengxian County in 2016 was highest and dropped dramatically in 2017. However, it increased again in 2018 and 2019. Similar trends could also be seen in other counties (Fig. 2). We surveyed the same villages over the past 4 years, although the same individuals may not have been tested each time. Most of infected individuals were treated after the survey, so the prevalence should be very low. In contrast, it remains high, suggesting that repeated infections are common.

## Discussion

The major findings of this study are that the prevalence of *C. sinensis* is very high while those of roundworm, whipworm, and pinworm are very low in Guangxi, China. The proportion of heavy infection with *C. sinensis* is significantly higher than those of STHs. The major human parasite in the south of Guangxi is STHs while that in its center is *C. sinensis*. The prevalence of *C. sinensis* in medical workers is significantly higher than in any other occupation. A strength of this study is that we carried out a cross-sectional study and a longitudinal surveillance study, which may provide not only the current prevalence of parasite infection but also the trend. Another strength is that the sample size is large, which may avoid sampling bias. A weakness of the study is that we have surveyed the same villages over the past 4 years, although the individuals investigated may not have been the same every time. Most of the infected individuals were treated after the survey, which may result in a biased prevalence.

In order to evaluate the effect of prevention and control measures and to provide data for policymaking, three national surveys of major human parasitic diseases were carried out throughout China in 1990, 2003, and 2015 [2]. Data from the three national surveys showed that the whole country’s average prevalences of hookworm, roundworm, and whipworm trended to be decreasing [2, 16]. Compared to these data, the prevalences of roundworm and whipworm here have become very low and also showed a decreasing trend. However, the prevalence of hookworm is much higher and with an increasing trend. Therefore, in the future, the resource of prevention and control should be focused on the affected group of older women in rural areas.

The average prevalence of *C. sinensis* in China was 0.47% in the third national survey [17]. According to the data in the second and third national survey, the prevalence of *C. sinensis* in Guangxi was 3.71% and 9.62%, respectively [18, 19]. Qian et al. compared the data collected from three parasitic disease surveys conducted in Hengxian County, Guangxi, China, during 1989-2011 and found that the prevalence of helminthiases decreased, while that of clonorchiasis increased, over time [20]. We found here that the prevalence of *C. sinensis* in the cross-sectional study is higher than that of third national survey, which was confirmed by the longitudinal surveillance study, suggesting that the prevalence of *C. sinensis* in Guangxi trends to be increasing.

*Clonorchis sinensis* is one of the most destructive parasitic worms in humans in China, Vietnam, Korea, and the Russian Far East. The worm is carcinogenic, inducing cholangiocarcinoma [21]. It is surprising to find that the prevalence of *C. sinensis* is the highest in medical workers, among all occupations. It seems unlikely that medical workers are not aware that eating raw freshwater fish may be harmful to their health. In some areas of Guangxi, such as Hengxian County, eating of raw fish is strongly encouraged to protect cultural traditions. Offering raw fish to guests is deemed a hospitable gesture. Economic development promotes *C. sinensis* infection because more people can afford to eat raw fish, not only at home but also in restaurants [9]. Meanwhile, many people could not resist the temptation of raw fish because it is very delicious after special preparation. Therefore, it is difficult for Guangxi to control *C. sinensis* infection.

Pinworm is a cosmopolitan parasite and one of the most common helminths infecting humans in temperate and cool climates, including developed countries. It is estimated that 1-33% of children are infected globally [22]. According to the data in the third national survey, the prevalence of pinworm in children aged 3 to 6 years is 3.4% and 8.7% in the whole country and Guangxi, respectively [17, 18]. We obtained similar results in this study. Pinworm infection often is endemic in overcrowded conditions, such as kindergartens and primary schools, due to the ease of transmission from infected to uninfected children [23]. Therefore, except where living conditions have improved greatly, regular pinworm screening and treatment is advised in Guangxi.

The rate of mixed infection with parasites varies geographically. It is as high as 34.6% in Ethiopia [24] but only 0.22% in Chongqing, China [25]. In this study, the dual infections rate is close to that in Chongqing. Living habits may be a risk factor for STHs infection [8]. Guangxi Zhuang Autonomous Region has 12 native ethnic groups: Zhuang, Han, Yao, Miao, Dong, etc. Each group has its own habits of eating, hygiene, etc. In this study, we found that the prevalence of hookworm, roundworm, whipworm, and pinworm are higher in ethnic minorities than the Han ethnic group, and this may be associated with their cultural habit. Therefore, health education should be promoted among these ethnic minorities.

In conclusion, Guangxi has achieved the national goal of China for the elimination of roundworm and whipworm infection. However, the prevalence of *C. sinensis* remains high and trends to be increasing. Enhanced health education should be focused on *C. sinensis* in Guangxi.

## Data Availability

The datasets used and/or analyzed during this study are available from the corresponding author on reasonable request.

## Abbreviations

STHs: Soil-transmitted helminths
*C. sinensis*: *Clonorchis sinensis*
EPG: Egg count per gram feces
SD: Standard deviation
CI: Confidence interval
